# Impact of Whole-Genome and Tandem Duplications in the Expansion and Functional Diversification of the F-Box Family in Legumes (Fabaceae)

**DOI:** 10.1371/journal.pone.0055127

**Published:** 2013-02-04

**Authors:** Daniel Bellieny-Rabelo, Antônia Elenir Amâncio Oliveira, Thiago Motta Venancio

**Affiliations:** Laboratório de Química e Função de Proteínas e Peptídeos, Centro de Biociências e Biotecnologia, Universidade Estadual do Norte Fluminense Darcy Ribeiro, Campos dos Goytacazes, Rio de Janeiro, Brazil; California State University Fullerton, United States of America

## Abstract

F-box proteins constitute a large gene family that regulates processes from hormone signaling to stress response. F-box proteins are the substrate recognition modules of SCF E3 ubiquitin ligases. Here we report very distinct trends in family size, duplication, synteny and transcription of F-box genes in two nitrogen-fixing legumes, *Glycine max* (soybean) and *Medicago truncatula* (alfafa). While the soybean FBX genes emerged mainly through segmental duplications (including whole-genome duplications), *M. truncatula* genome is dominated by locally-duplicated (tandem) F-box genes. Many of these young FBX genes evolved complex transcriptional patterns, including preferential transcription in different tissues, suggesting that they have probably been recruited to important biochemical pathways (e.g. nodulation and seed development).

## Introduction

Covalent modification of proteins by the attachment of ubiquitin (Ub)-like polypeptides (e.g. ubiquitin, SUMO, Urm1) a pervasive post-translation modification that can be destabilizing (e.g. lysine 48 polyubiquitination) or non-destabilizing (e.g. sumoylation or lysine 63 monoubiquitination) [1]. Initially thought to be a eukaryotic innovation, antecedents of the ubiquitin conjugation machinery have been characterized in several prokaryotic genomes [2]–[5]. Ub/Ubl conjugation result from the concerted activity of three key of enzymes (i.e. E1, E2 and E3), aided by several regulatory proteins and the proteasome system [6]. After the proteolytic processing of the Ub/Ubls from longer precursors, E1s catalyze the ATP-dependent adenylation of the C-terminal carboxylate, followed by a trans-thiolation of the Ub/Ubl to the active cysteine of the E2 [1], [6]. E2s can directly transfer the Ub/Ubl to the substrate with the aid of a RING-finger (or related) domain E3 ligase [7]. Alternatively, they can trans-thiolate the Ub/Ubl to HECT ligases, that catalyze the ultimate modification of the substrates [8]. E3s frequently harbor other subunits, such as F-box (FBX) proteins, cullins and POZ domain proteins. Ub is recycled at the proteasome by JAB-domain de-ubiquitinating metallopeptidases (DUBs) [8]. Other peptidases also exert regulatory roles in removing Ub/Ubls from several substrates, playing important roles in the Ub/Ubls signaling pathways [9], [10].

FBX proteins have a N-terminal Skp1-binding FBX domain, followed by a variable C-terminal region that confers substrate specificity to SCF (Skp1-Cullin1-F-box) E3 ligases. FBX genes are typically very numerous across several eukaryotic genomes, being involved in various biological processes, from hormone signaling to defense mechanisms [11]–[15]. Notable examples of FBX proteins in plant physiology are Tir1, Coi1 and Ein3, respectively involved in IAA (auxin), jasmonate and ethylene signaling cascades [16]. The FBX family is among the largest gene family in plants [17] and its size can be remarkably distinct across lineages, with no obvious correlation with evolutionary distance, genome size, organismal complexity and niche [18], [19].

Lineage-specific gene expansions (LSEs) result from single-gene, segmental, chromosomal or even whole genome duplications (WGDs), followed by preferential retention of some families [20]–[22]. Although potentially deleterious [23], WGD (i.e. polyploidization) is much more common in plants than in other lineages, being considered a major driver of speciation, diversification and adaptation to the most different niches [24], [25]. It has been hypothesized that a WGD was critical in the emergence of nodulation in legumes (Fabaceae or Leguminosae), the third largest angiosperm family [26], [27].

In the present study we explore aspects related to the emergence and functions of FBX genes in two recently sequenced legume genomes [26], [28]. Specifically, we show that disparate mechanisms can severely impact the size and genomic context of the FBX genes in short periods of time. For example, while many *Glycine max* FBX content emerged from segmental duplications, *Medicago truncatula* shows a high prevalence of FBX gene duplications in tandem. Moreover, several tandemly-duplicated FBX genes have evolved strong differential transcriptional profiles across different tissues, indicating their involvement in tissue-specific transcription, which might be a result of recent recruitment to important biological functions (e.g. nodulation and seed development and maturation).

## Results and Discussion

As a first step to understand the evolution of the FBX family in legumes, we used sensitive sequence analysis to scan the genomes of two nitrogen-fixing legumes, *Glycine max* (soybean) and *Medicago truncatula*. *Arabidopsis thaliana* (Eurosids II) and *Vitis vinifera* (grape) (basal rosid) were included as outgroups. *A. thaliana* is the most suitable model plant for molecular biology experiments, while grape is a valuable species in comparative genomics studies because its genome is apparently free of recent whole-genome duplications (WGD) and massive genome-wide rearrangements [29]. We found remarkably variable FBX family sizes across these species, which is a direct consequence of lineage-specific gains and losses. Specifically, we found FBX repertoires of 480 (*G. max*), 913 (*M. truncatula*), 688 (*A. thaliana*) and 147 (*V. vinifera*) genes. These results are generally consistent with that reported by a recent study of the FBX family in several plants [19].

**Figure 1:**
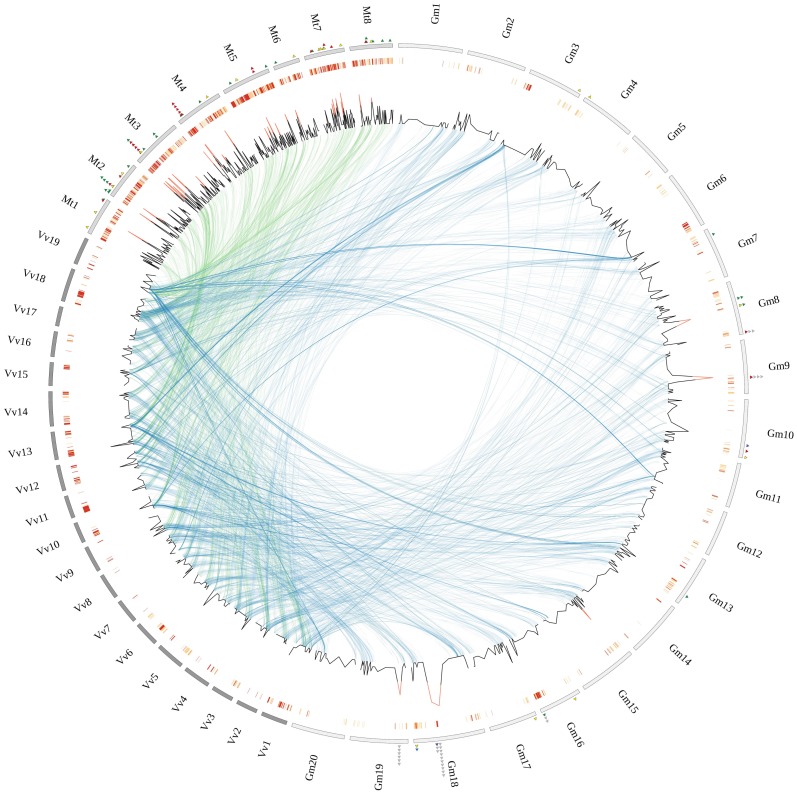
Homologous segments between *V. vinifera* and two legume species, *G. max* and *M. truncatula*. The outer circle shows numbered chromosomes of each species in gray (*M. truncatula*, Mt), light gray (*G. max*, Gm) and dark gray (*V. vinifera*, Vv). Local duplications are represented in the second outer circle, where red denotes higher density of tandem duplications in a particular region. The line plot illustrates the number of FBX genes in each interval of 100 genes. If 5 or more FBX genes are present in a given region, the peak is colored in red. Internal arcs connect syntenic regions between *V. vinifera*/*G. max* (blue) and *V. vinifera*/*M. truncatula* (green). Colored triangles represent tandemly-duplicated FBX genes with preferential expression in late-development seeds (green), late embryogenesis seeds (red), nodules (yellow). For Gm: no detectable transcription (gray), apical meristem (green), nodule (blue), flower (yellow) and leaves (purple).

The highly variable FBX content observed in two closely-related legumes stimulated us to explore the genomic architecture of this family. Firstly, we sought to investigate the prevalence of FBX genes in syntenic regions, which is suggestive of architectural conservation in ancient genomes (Figure S1). The statistical significance of our results was assessed by inspecting the proportion of FBX in 10,000 simulated sets of syntenic regions (see methods for details). Again, here we found striking differences between closely-related species – out of the 480 *G. max* FBX genes, 186 (∼38.8%) are located in syntenic blocks encompassing 74/147 (50.3%) *V. vinifera* FBX counterparts. Moreover, 95.7% (178 genes) of the soybean FBX genes syntenic to grape map to segmentally duplicated regions, implying that the two WGD events that happened after the split of basal rosids (e.g. *V. vinifera*) and the ancestral of Eurosids I and Eurosids II clades [30] significantly contributed to the soybean FBX gene complement. Conversely, in spite of having shared one of these WGD events in its natural history, only 9.4% of the *M. truncatula* FBX genes (86 genes) are syntenic to *V. vinifera* (Figure S1). In addition, *M. truncatula* has virtually doubled its FBX gene complement after the split with soybean (see below) (Figure 1).

**Figure 2:**
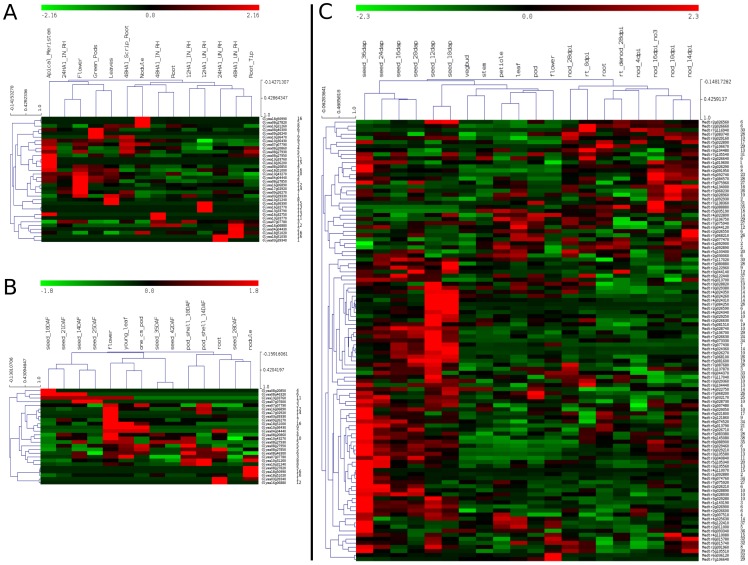
Transcriptional profiles of tandemly-duplicated FBX genes in *G. max* and *M. truncatula* in different tissues. Normalized transcriptional levels were obtained from Severin at al [30] (A) and Libault et al [31] (B) (*G. max*) and Benedito et al (*M. truncatula*) (C) [39]. For each independent study, gene expression values were standardized using Z-score and clustered with Hierarchical Clustering (MeV package). Numbered labels in the right refer to tandem FBX arrays (i.e. if two genes have the same number, they are very close to each other in the genome). These labels are qualitative and thus there is no correlation between label number and genomic closeness of the tandem FBX arrays.

It is clear from our work and others [19], [26] that tandem gene duplication is the main evolutionary force underlying the complexity of the FBX gene family in *M. truncatula* –53.8% of the FBX genes (491 of 913) in *M. truncatula* map to tandem arrays (Figure 1; Table S1). A remarkably FBX-dense region is located in *M. truncatula* chromosome 3, encompassing 30 FBX genes across ∼368 Kb. Several FBX genes in this region are not only transcriptionally active, but also preferentially expressed in particular tissues (Figure 1 and 2). Due to incomplete platform coverage, new genome assembly releases and potential cross-hybridization problems, only 109 of the 491 *M. truncatula* tandem FBX genes had valid microarray probe sets assigned. The global transcriptional profile of these 109 locally duplicated FBX genes revealed three major clusters: late embryogenesis (heart stage) and transition phase; late seed development (seed filling); and nodules (mature and nitrogen-fixing) (Figure 2). Interestingly, the nodule transcriptional FBX cluster has genes from recent independent local FBX duplications (e.g. *Medtr2g091950*, *Medtr4g134000* and *Medtr7g138360*) that are not only highly transcribed, but also responsive to NO3 treatment (Figure 2 and Figure S2), suggesting that they might play important regulatory roles in nitrogen fixation. In addition, several tandemly duplicated FBX genes are involved in late embryogenesis, seed filling and maturation (Figure 2), suggesting that they drive the degradation of specific enzymes and impact the protein content in mature seeds. Alternatively, several FBX mRNAs available in late seed development could be stored in dry seeds to be used during the early germination steps. RNA-Seq data for *M. truncatula* will certainly improve the coverage of the whole *M. truncatula* transcriptome and allow comparative studies with the soybean transcriptional maps.

As opposed to *M. truncatula*, only 15% (72 of 480) of the Gm FBX genes originated by tandem gene duplications. A remarkably FBX-dense region can be found in the soybean chromosome 18, harboring 16 FBX and at least 5 potentially inactive FBX genes (i.e. genes that have lost the FBX domain but retained similarity with other FBX genes) along ∼497 Kb (39,737,479 to 40,234,206) (Figure 1). Interestingly, neither of the soybean transcriptomes analyzed here [30], [31] detected the transcription of these FBX genes (Table S1), implying that they are either inactive or transcribed in specific conditions yet to be studied (e.g. chemical and pathogen stress). We found that 64.29% (36/56) of the remaining *G. max* tandemly-repeated FBX genes are transcriptionally active in at least one tissue/condition (Figure 2). Moreover, while some neighboring genes retained similar transcriptional patterns after duplication, others are clearly divergent (Figure 2; Table S1). For example, *Glyma18g51020*, *Glyma18g50990* and *Glyma18g51000* are neighbors in chromosome 18; while the latter gene is mainly transcribed in aerial parts, the two former are strongly transcribed in nodules and might be involved in regulating processes related to nitrogen fixation. This transcriptional divergence suggests a recent functional diversification in this FBX array, a trend that is also observed in many other locally duplicated FBX genes in *G. max* and *M. truncatula* (Figure 2). Interestingly, other individual FBX genes from different tandem arrays that have also evolved differential transcription in nodules in both independent transcriptome studies (e.g. *Glyma08g27820* and *Glyma10g31260*) (Figure 2), strongly suggesting that SCF-mediated ubiquitination might play critical roles in regulating the degradation of specific substrates to control nitrogen fixation in soybean.

Taken together, the results presented here indicate that the FBX inventory can be highly variable between closely related species. Many of such expansions and deletions in the recent natural history of legumes probably happened through genomic drift [18], [19], providing a source of variation for for natural selection to act upon. Strong transcriptional evidence (Table S1) and the integrity of gene structures suggest that many locally duplicated FBX genes have been recruited to biochemical pathways involved in critical legume traits (e.g. nodulation and seed maturation). Although it has been shown that miRNAs are key regulators of FBX-mediated signaling processes in plants, it is possible that they play some role in the divergent transcriptional profiles observed for some tandemly repeated FBX [32]. The results presented here suggest several interesting gene candidates for additional biochemical experiments, aiming to understand their precise roles and functional diversification in legumes.

## Materials and Methods

The predicted protein sequences of *M. truncatula* [26], *G. max* [28], *A. thaliana* [33] and *V. vinifera* [29] were downloaded from the Phytozome FTP server (http://www.phytozome.net/). Protein domain architectures were computed using the HMMer package [34] and the Pfam domain database [35]. Three domains from the Pfam F-box clan (i.e. F-box, F-box-like, F-box-like_2) were used to detect the FBX proteins from each genome, using an e-value threshold of 1.0 and 50% of the FBX domain aligned. This high e-value cutoff is required to avoid false-negative predictions, as previously discussed by Hua et al [19]. The domain coverage parameter was included in our analysis to control for false-positives.

BLASTp [36] searches were conducted using the predicted proteomes of all four species (all vs al; E-value ≤0.01). Synteny analysis, local (tandem) and segmental duplications were identified using DAGchainer [37]. Proteins with unknown genomic loci were not used in this analysis. DAGchainer default parameters were used, except for requiring the alignment of 4 genes to define a syntenic block (i.e. –A parameter). Specific parameters were set to detect tandem and segmental duplications in each genome (-T and –I, respectively). Ideograms were created using Circos [38]. To evaluate if the FBX genes are preferentially located inside or outside syntenic across pairwise comparisons, gene labels were shuffled to build 10,000 synteny files for each comparison. In cases where segmental duplications resulted in one-to-many or many-to-one relationships, the occurrences of shuffled labels were distributed accordingly. The expected frequency of FBX genes resulting from the simulations was then compared to the observed frequency of FBX genes in the real data. A similar procedure was applied to interrogate the frequencies of FBX genes in tandem duplications.

*G. max* [30], [31] and *M. truncatula* [39] transcriptional data were downloaded and standardized using the z-score transformation. The soybean datasets were generated using RNA-Seq technologies and normalized values were downloaded from the original articles. Conversely, *M. truncatula* transcriptional data were generated using an Affymetrix ™ microarray platform, which required us to update valid identifiers, remove genes with deprecated identifiers and potentially cross-hybridizing probesets. Standardized transcriptional data were then visualized and clustered with the MeV software [40].

## Supporting Information

Figure S1 **Distribution of transcriptional values of all** ***M. truncatula*** **genes represented in the microarray platform used by Benedito et al** [**39**] **.** The logarithm of the highest expression value of each gene was used to compute the density estimates. Represented tissues are: seeds (black), petiole (blue), stem (red), apical meristem (brown), flower (magenta), pods (yellow), roots (orange) and nodules (purple). Red and black tick marks represent FBX genes located inside or outside tandem arrays, respectively.
(TIF)

Figure S2 **The table represents the number of FBX genes in syntenic regions between each pair of species.** Inside parenthesis is the mean number of FBX genes in syntenic regions observed in the simulated synteny maps, followed by the standard deviation. Graphs show the number of FBX genes in the simulated synteny maps. Each fine red line refers to one simulation.
(TIF)

Table S1 **Tandemly repeated FBX genes transcribed in at least one tissue of** ***M. truncatula*** **and** ***G. max*** **.** For *G. max* we included all tandemly-repeated FBX genes reported as transcribed by the authors who generated the data [30], [31]. For *M. truncatula* we included all the tamdem FBX genes with normalized transcription greater than 10.0 [39]. Due to the incomplete coverage of the *M. truncatula* microarray platform, not all the tamdemly-repeated FBX genes were interrogated for this species.
(XLS)
