# Supplementary figures and images for: Impact of Whole-Genome and Tandem Duplications in the Expansion and Functional Diversification of the F-Box Family in Legumes (Fabaceae)
Source: PLoS One. 2013 Feb 4;8(2):e55127. doi: 10.1371/journal.pone.0055127 (PMC3563651; doi:10.1371/journal.pone.0055127)

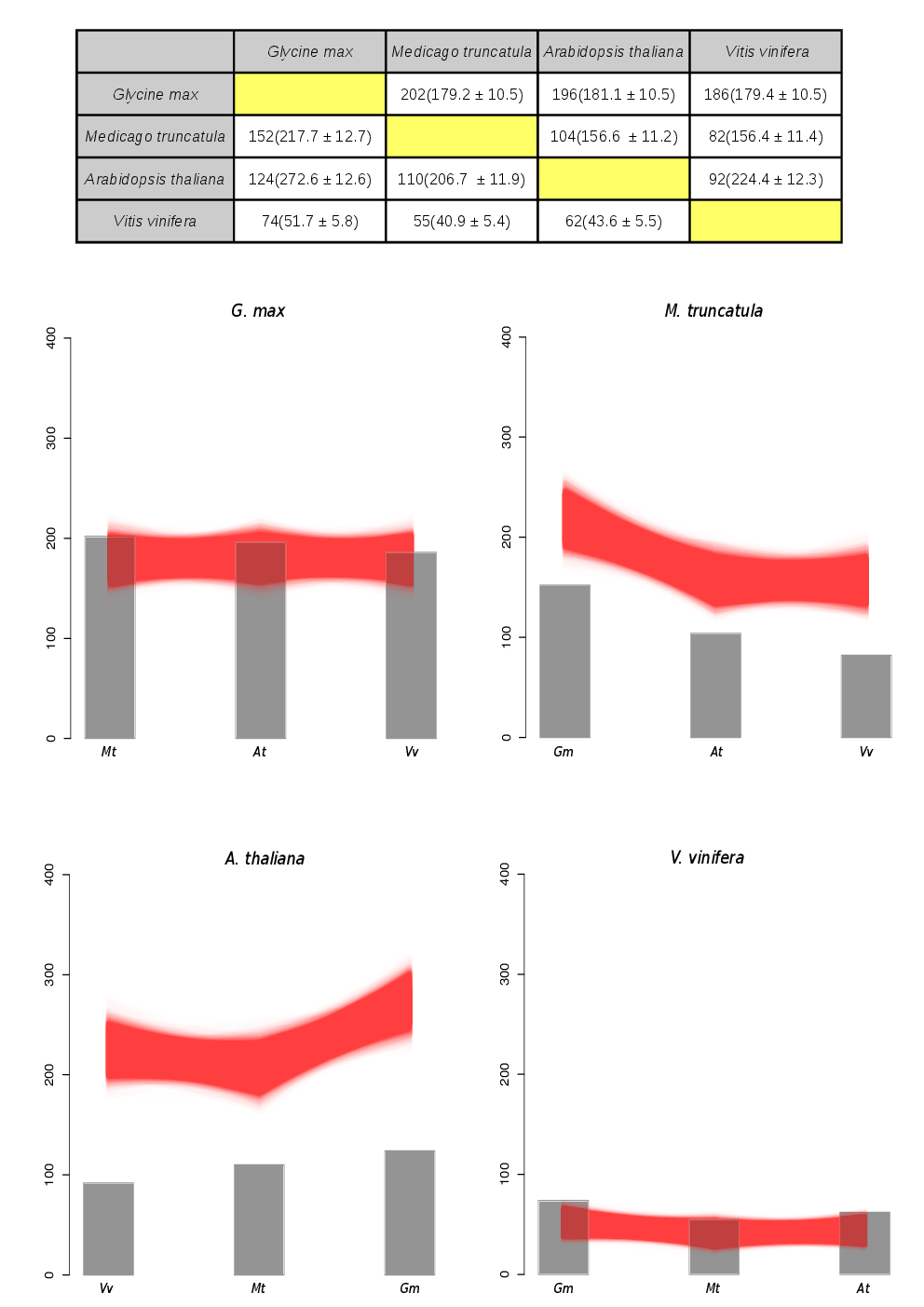

Supplement: Figure S1 — Distribution of transcriptional values of all M. truncatula genes represented in the microarray platform used by Benedito et al [39] . The logarithm of the highest expression value of each gene was used to compute the density estimates. Represented tissues are: seeds (black), petiole (blue), stem (red), apical meristem (brown), flower (magenta), pods (yellow), roots (orange) and nodules (purple). Red and black tick marks represent FBX genes located inside or outside tandem arrays, respectively. (TIF) [file pone.0055127.s001.tif]

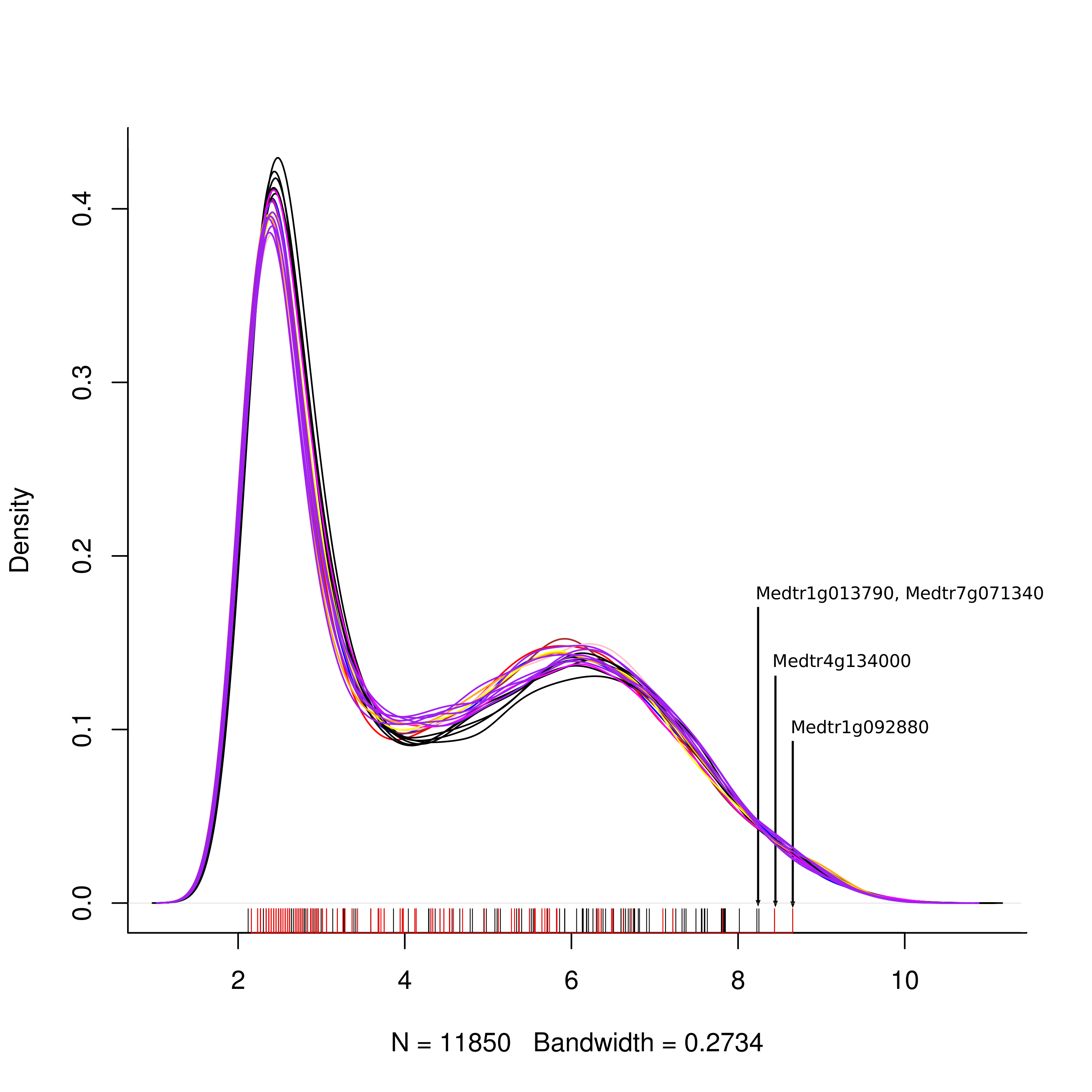

Supplement: Figure S2 — The table represents the number of FBX genes in syntenic regions between each pair of species. Inside parenthesis is the mean number of FBX genes in syntenic regions observed in the simulated synteny maps, followed by the standard deviation. Graphs show the number of FBX genes in the simulated synteny maps. Each fine red line refers to one simulation. (TIF) [file pone.0055127.s002.tif]
